# Endogenous DNA breaks: γH2AX and the role of telomeres

**DOI:** 10.18632/aging.100025

**Published:** 2009-02-17

**Authors:** Peggy L. Olive

**Affiliations:** Medical Biophysics Department, British Columbia Cancer Research Center, Vancouver, B.C., Canada V5Z 1L3

**Keywords:** DNA damage, cancer, cell cycle, histone, DNA repair

DNA double-strand breaks (DSBs) rarely form in living cells but can be deadly. A single unrepaired DSB will kill a yeast cell deficient in recombination [1].  Unrepaired DSBs lead to chromosome damage and are associated with aging and cancer.  Until a few years ago, methods used for their detection relied exclusively on physical techniques such as pulsed field gel electrophoresis that measure changes in the size of DNA molecules.  Unfortunately, these methods are insensitive, typically recognizing 50 or more breaks per mammalian cell and necessitating the use of lethal exposures to X-rays or radiomimetic drugs.    In 1998, Bonner and colleagues reported that phosphorylation of H2AX, a minor nucleosomal histone protein, occurred at sites of DSBs [2].  This process is unique in that hundreds of molecules surrounding each break become phosphorylated as the signal propagates away from the break site; development of antibodies against the serine-139 phosphorylated form (called γH2AX) allowed microscopic detection of individual DSBs [3]. This discovery revolutionized the ability to detect DSBs and provided a unique tool to examine processes involved in DNA damage signalling.  Applications of γH2AX as an indicator of response to radiation and drugs soon followed [4].

Sensitive detection of drug- and radiation-induced DSBs using γH2AX requires a low endogenous expression of γH2AX.  Similarly, applications of γH2AX in DNA damage signalling are dependent on low endogenous levels of the phosphorylated form.  In most normal primary human cells, γH2AX foci are relatively  rare  so  that  DSBs  can be detected by nonlethal radiation doses in the mGy range [5].  However, γH2AX foci are observed in cells undergoing meiosis or V(D)J recombination, as well as senescent cells and apoptotic cells.  In each of these cases, a convincing argument can be made that DSBs underlie the formation of γH2AX foci.  It is more difficult to explain large numbers of endogenous γH2AX foci seen in many tumors cells, or the variability in foci numbers between different tumor cell lines [6].  As physical methods lack the sensitivity to confirm that these foci signify true breaks, the possibility remains that either some tumor cells contain large numbers of DSBs or there are other explanations for endogenous foci. In either case, endogenous foci are a problem because they reduce the sensitivity and specificity for detecting exogenously produced breaks.

In this issue of *Aging,* Nakamura et al. examine the possibility that endogenous foci in tumor cells are associated with telomeres.  Telomeres are composed of repeat DNA sequences that constitute the natural ends of chromosomes. Normally, chromosome ends are protected by telomere associated proteins like TRF2 and by the formation of a looped structure created by the repeat sequences [7].  However, when left "uncapped", chromosome ends will provide a signal for H2AX phosphorylation.  In aging mice and during cellular senescence when telomeres erode and become critically short, γH2AX foci are formed [8,9]. Uncapped telomeres, created by inhibition of TRF2, have been shown to associate with several DNA damage response factors, including γH2AX and 53BP1 [10].  Although telomerase is activated in most tumor cells to counteract telomere erosion and confer immortality, telomeres in tumor cells can still be abnormally short and dysfunctional.  Telomere dysfunction, whether resulting from erosion, breakage-fusion-bridge cycles, or other mechanisms, has been associated with chromosome instability and cancer progression [11].

Can telomere shortening also explain the presence of excessive endogenous γH2AX foci in many tumor cell lines?  This is reasonable because telomere erosion will trigger a DNA damage response yet would not result in any additional DSBs.  Warters et al. [12] suggested that dysfunctional telomeres could be responsible for the endogenous γH2AX foci they observed in several melanoma cell lines; in their studies, some co-localization occurred between γH2AX foci and TRF1. In this issue, Nakamura et al. have asked this question directly by co-staining metaphase tumor cells with antibodies against γH2AX together with telomere-FISH staining; they used this approach previously to confirm the importance of telomeric damage in γH2AX foci that developed in senescing normal cells (i.e., cells lacking telomerase) [9].   Now using tumor cells, they find up to 4 times more γH2AX foci at telomeric than non-telomeric regions.  Moreover, γH2AX foci formed preferentially at FISH-negative ends.  This result strongly implicates a role for eroded telomeres in stimulating H2AX phosphorylation.  In addition, variability in numbers of endogenous γH2AX foci seen among 5 different tumor cell lines could be explained by differences in the proportion of telomere associated foci.  This observation led Nakamura et al. to examine telomerase activity in these 5 cell lines.  Consistent with the importance of telomeres, tumor cells with more endogenous foci per metaphase also showed lower telomerase activity.

Is it possible that γH2AX foci at chromosome ends do more than mark the presence of uncapped or dysfunctional telomeres?  For the foci that formed at telomeres in senescing cells, H2AX did not appear to play a role in senescence since H2AX-null mice have a normal life span [9].  Perhaps a similar conclusion could be made regarding endogenous foci in tumor cells; excessive endogenous foci appear to have few functional consequences in terms of clonogenicity or proliferation rate. However, Yu et al. [6] showed that tumor cells with more endogenous foci exhibited greater chromosomal instability, an observation that can now be explained by differences in telomere dysfunction. This also ties in nicely with the behavior of telomeres because the organization of telomeres in tumor cells turns out to differ from that of normal cells.  In interphase tumor cells, telomeres can form various-sized aggregates whereas in normal cells, a non-overlapping telomere pattern is observed [13].  Although the process of telomere aggregation in tumor cells is poorly understood, it has been associated with genomic instability [14].  Perhaps clustering of telomeric foci in these telomeric aggregates contributes to the variability in γH2AX foci size and number seen in untreated tumor cells.

Although damage at telomeres explains most of the endogenous foci in these 5 tumor cell lines, non-telomeric associated foci were also observed in metaphase cells.  In fact, half of the foci in one of the lines, HCT116, were non-telomeric.  Although some of these foci could represent telomere fusion events, their origin remains in question. Within each of the 5 cell lines, many cells exhibited in excess of 20 foci, not all of which are likely to represent DSBs or to be telomere associated.  Timing is critical. If a DSB does form transiently at the end of a chromosome, the γH2AX focus may persist for a long time after the break is rejoined, even through cell division.  DNA damage signaling in terms of H2AX phosphorylation differs in normal cells versus tumor cells.  Loss of p53 has no effect on rate of DSB rejoining but does result in a higher endogenous expression of γH2AX and longer retention of radiation-induced γH2AX foci [6]. Determining whether a γH2AX focus marks the site of a current or past DSB is not a simple matter.  To add to the complexity, a physical break is apparently not necessary for phosphorylation of H2AX.  Simply presenting NBS1 molecules (a DNA repair protein that co-immunoprecipitates with γH2AX) on a length of chromatin provides an adequate signal to activate H2AX phosphorylation at that site [15].

The current results of Nakamura et al. are based on the most sensitive measure of γH2AX induction, immuno-cytochemical analysis of individual foci.  Therefore the physical size of individual γH2AX foci is critical.  Microscopic resolution is limited to about 0.2 microns, so foci below this size will not be detected. However, even in the absence of microscopically visible foci or exogenous damage, some H2AX is phosphorylated.  This amount is higher in cells synthesizing DNA; S phase cells exhibit γH2AX foci which are usually discounted as they are much smaller and do not associate with DNA damage response proteins like 53BP1 [16].  There are exceptions, however, and mouse pluripotent embryonic stem cells express on average 100 endogenous γH2AX foci that cannot be distinguished from radiation-induced foci on the basis of size or intensity.   Banáth et al. [17] suggested that the large foci in these cells could be explained by histone  hyperacetylation and abundant chromatin remodeling complexes, both of which are known to enhance the size of γH2AX foci.  So if chromatin organization influences foci size, at least some of the endogenous foci in these tumor cells might be a result of local changes in chromatin structure that allow small foci to grow larger and become microscopically visible.   Genomic instability and chromatin anomalies go hand in hand.

Nakamura et al. raise the intriguing possibility that γH2AX foci could provide a "biomarker" to indicate which tumor cells are more likely to respond to telomerase inhibition.  Any drug that inhibits telomerase activity and leads to telomere erosion or dysfunction should result in γH2AX foci, as shown previously by Takai et al. when TRF2 activity was blocked [10].  Having a simple tool to evaluate the efficacy of telomerase inhibitors should prove very useful in the development of a tumor targeted therapy.

## References

[1] Frankenberg-Schwager M, Frankenberg D (1990). DNA double-strand breaks: their repair and relationship to cell killing in yeast. Int J Radiat Biol.

[2] Rogakou EP, Pilch DR, Orr AH, Ivanova VS, Bonner WM (1998). DNA double-stranded breaks induce histone H2AX phosphorylation on serine 139. J Biol Chem.

[3] Rogakou EP, Boon C, Redon C, Bonner WM (1999). Megabase chromatin domains involved in DNA double-strand breaks In vivo. J Cell Biol.

[4] Bonner WM, Redon CE, Dickey JS (2008). gammaH2AX and cancer. Nat Rev Cancer.

[5] Lobrich M, Rief N, Kuhne M (2005). In vivo formation and repair of DNA double-strand breaks after computed tomography examinations. Proc Natl Acad Sci U S A.

[6] Yu T, MacPhail SH, Banath JP, Klokov D, Olive PL (2006). Endogenous expression of phosphorylated histone H2AX in tumors in relation to DNA double-strand breaks and genomic instability. DNA Repair (Amst).

[7] de Lange T (2002). Protection of mammalian telomeres. Oncogene.

[8] Sedelnikova OA, Horikawa I, Zimonjic DB, Popescu NC, Bonner WM, Barrett JC (2004). Senescing human cells and ageing mice accumulate DNA lesions with unrepairable double-strand breaks. Nat Cell Biol.

[9] Nakamura AJ, Chiang YJ, Hathcock KS (2008). Both telomeric and non-telomeric DNA damage are determinants of mammalian cellular senescence. Epigenetics Chromatin.

[10] Takai H, Smogorzewska A, de Lange T (2003). DNA damage foci at dysfunctional telomeres. Curr Biol.

[11] Murnane JP (2006). Telomeres and chromosome instability. DNA Repair (Amst).

[12] Warters RL, Adamson PJ, Pond CD, Leachman SA (2005). Melanoma cells express elevated levels of phosphorylated histone H2AX foci. J Invest Dermatol.

[13] Mai S, Garini Y (2006). The significance of telomeric aggregates in the interphase nuclei of tumor cells. J Cell Biochem.

[14] Goldberg-Bittman L, Kitay-Cohen Y, Quitt M (2008). Telomere aggregates in non-Hodgkin lymphoma patients at different disease stages. Cancer Genet Cytogenet.

[15] Soutoglou E, Misteli T (2008). Activation of the cellular DNA damage response in the absence of DNA lesions. Science.

[16] McManus KJ, Hendzel MJ (2006). ATM-dependent DNA damage-independent mitotic phosphorylation of H2AX in normally growing mammalian cells. Mol Biol Cell.

[17] Banáth JP, Banuelos CA, Klokov D, Macphail SM, Lansdorp PM, Olive PL (2009). Explanation for excessive DNA single-strand breaks and endogenous repair foci in pluripotent mouse embryonic stem cells. Exp Cell Res.

